# Plant–Fungi Mutualism, Alternative Splicing, and Defense Responses: Balancing Symbiosis and Immunity

**DOI:** 10.3390/ijms26115197

**Published:** 2025-05-28

**Authors:** Xiaoqiong Zhao, Mehtab Muhammad Aslam, Moxian Chen, Debatosh Das

**Affiliations:** 1 Key Laboratory of Plant Resource Conservation and Germplasm Innovation in Mountainous Region (Ministry of Education), Institute of Agro-Bioengineering, College of Life Sciences, Guizhou University, Guiyang 550025, China; 13124670312@163.com; 2 State Key Laboratory of Green Pesticides, Key Laboratory of Green Pesticide and Agricultural Bioengineering, Ministry of Education, Center for Research and Development of Fine Chemicals, Guizhou University, Guiyang 550025, China; 3 Division of Plant Sciences & Technology, College of Agriculture, Food and Natural Resources (CAFNR), University of Missouri, Columbia, MO 65201, USA; mehtabmuhammadaslam@yahoo.com; 4 Natural Products Utilization Research, U.S. Department of Agriculture, Agricultural Research Service, Oxford, MS 38677, USA

**Keywords:** alternative splicing, molecular mechanisms, plant–fungal interactions, post-transcriptional regulation, small molecules

## Abstract

Alternative splicing (AS) is the process of RNA maturation in eukaryotes, which is essential for post-transcriptional regulation. The transcripts produced by AS can encode distinct protein isoforms and contribute to the regulation of eukaryotic growth and development in response to a changing environment, and they are crucial in plant–fungal interactions. Plant–fungal symbiosis is one of the most significant biotic interactions in the biosphere. The symbiotic association of fungi not only improves plant growth and resistance but has potential significance for endangered species conservation and reproduction. Alternative splicing is involved in regulating symbiotic responses to host immune signals, regulating the host–symbiont contact, and initiating signaling during symbiosis. In recent years, mRNAs research has been progressing rapidly, and AS is an important post-transcriptional regulator that requires further investigation. However, while AS has been widely studied in mammalian disease research, very limited studies are available on the regulation of AS in plant–fungal symbiosis and their role in benefiting the interacting partners. In this review, we provide an overview of our existing knowledge about AS in symbiotic plant–fungal relationships and discuss potential hotspots for future investigation to expand our understanding of plant–fungal interactions.

## 1. Introduction

Plant–fungi [1] symbiosis ensures mutual resource exchange, optimizing nutrient transfer. Plants provide carbohydrates to fungi, which reciprocate with soil-derived nutrients, enhancing plant adaptation to environmental stress and agricultural productivity [2]. Studies have demonstrated the reinforcement of plant–fungi symbiosis. For example, *Serendipita indica* secretes acid phosphatases, enhancing phosphorus availability for host plant growth [3], and enhances host plant performance and stress resistance [4]. The endophyte *S. indica* forms beneficial root symbioses. Additionally, arbuscular mycorrhizal fungi reduce seedling dependency on photosynthesis [5] and bolster resistance to environmental stresses such as drought and heavy metal exposure [6].

Alternative splicing (AS) [7] is a post-transcriptional regulatory mechanism unique to eukaryotes, generating diverse mRNA isoforms by spliceosomes. AS, along with gene replication, contributes significantly to proteomic functional diversity [8], influences species complexity and adaptation, and is crucial in plant–fungal symbiosis evolution and biological adaptability [9]. AS events, classified into four types, including exon skipping, selective 3′ splice sites (SS), alternative 5′ SS, and intron retention. Pre-RNA can form various spliceosomes through splicing, which can directly participate in regulating plant gene expression as transcription factors [10,11], form other functional receptor proteins to participate in plant signal transduction [12], or form nonfunctional proteins to silence certain metabolic functions [13].

The molecular mechanisms underlying plant–fungal symbiosis have garnered increasing research attention. This review systematically summarizes the regulatory roles of AS in plant–fungal interactions and the associated analytical tools (Table 1). Current studies employ an integrated multiomics approach: Illumina short-read sequencing enables genome-wide AS screening, while PacBio long-read sequencing resolves complex isoform structures. Following GMAP alignment, SUPPA2 quantifies AS events, with DRIMSeq and DESeq2 analyzing transcript-level and gene-level expression differences, respectively, and IsoformSwitchAnalyzeR evaluating functional consequences. Combined with experimental validation and expression profiling, this strategy has successfully elucidated the dynamic regulatory networks of AS during symbiotic processes.

**Figure 1:**
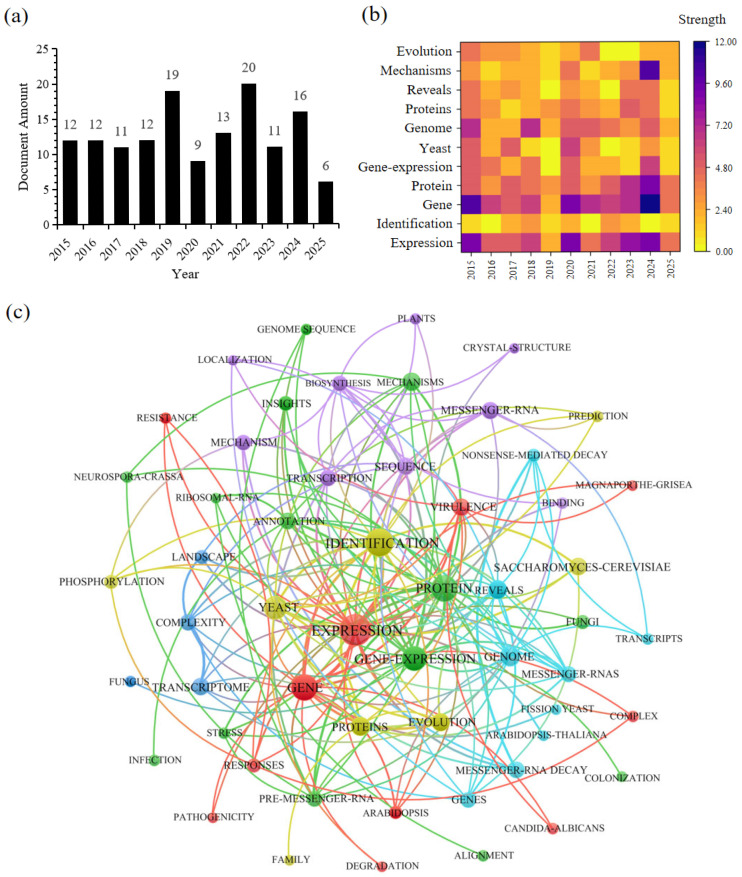
Bibliometric analysis of fungi and alternative splicing based on the core database of the Web of Science. (**a**) Histogram of the number of documents with the keywords “Fungi” and “Alternative splicing”; (**b**) heatmap of bibliometric analysis with “Fungi”, “Alternative splicing”, “Expression”, “Identification”, “Gene”, “Protein”, “Gene-expression”, “Yeast”, “Genome”, “Proteins“, “Reveals”, “Mechanisms”, and “Evolution” as keywords in the Web of Science core database; (**c**) network diagram of bibliometric analysis of fungi and alternative splicing research based on the Web of Science core database.

A bibliometric analysis of plant–fungal symbiotic AS research (2015–2025) from the Web of Science Core Collection (Figure 1) revealed that current studies primarily focus on AS regulation in nonmodel plants, signal recognition mechanisms, and comparative transcriptomics. The field has exhibited two major paradigm shifts: from single-gene analysis to microbial interaction networks, and from descriptive research to functional validation and molecular design applications. This analysis establishes an empirical foundation for understanding knowledge evolution and technological innovation in symbiotic AS research while pioneering new avenues for developing novel agricultural biotechnologies.

## 2. Classification of Symbiotic Fungi and Their Ecological Functions

### 2.1. Diversity of Symbiotic Fungi

Symbiotic fungi, such as arbuscular mycorrhizal (AM) fungi [9], form crucial associations with most land plants, colonizing roots and enhancing nutrient access, particularly access to phosphorus, in exchange for plant sugars. These interactions, found in approximately 72% of plant–fungi symbioses, are essential for plant growth and health. Other mycorrhizal types include ectomycorrhizae (ECM), ericoid mycorrhizae (ERM), and orchid mycorrhizae (ORM) [23]. While most vascular plants form mycorrhizae, about 8% do not, with various forms of nonmycorrhizal and mixed associations making up the remainder [23]. These symbioses are key for nutrient uptake and protection against stresses through secondary metabolites [24].

Ectomycorrhizal fungi (EMF) form a key symbiotic relationship with the roots of forest trees [25], creating a nutrient exchange interface outside plant cells [9]. Unlike other mycorrhizae, EMF diversity is linked to the host plant’s basal area and tree species diversity. Hydrophilic EMFs can redistribute nutrients among hosts in nutrient-rich soil areas [26]. Some EMFs facilitate high-altitude pine invasions [27], illustrating how microbial symbionts, along with plant traits and environmental factors, contribute to plant invasion success.

Ericoid mycorrhizae (ERM) penetrate the root cell walls of specific plant families such as *Ericaceae* and *Diapensiaceae* [25], forming dense mycelial coils within epidermal cells in the absence of specialized adhesion structures [9]. Unlike ectomycorrhizal fungi (ECM), ERM fungi directly invade host cells and produce biofilms and symbiotic interfaces [25]. These fungi regulate plant growth by producing phytohormones, enhancing resistance to abiotic stress, and degrading organic matter, thereby contributing to soil enrichment with secondary compounds. ERM are mainly associated with certain fungal orders such as *Helotiales* and potentially other *Agaricomycates* [23].

Orchid mycorrhizae (ORM) make up the second most common type of mycorrhizae. ORM are formed during seed germination in postembryonic tuber-shaped structures, where the mycelium passes through epidermal hair cells, reaches thin-walled cortical tissue, and ultimately forms large hyphal coils at the symbiotic interface [9]. It has been shown that orchids usually form mycorrhizae in symbiosis with fungi belonging to the order Stramonium, especially *Cantharellales*, and that ORM fungi promote orchid germination and protocol development by inducing an anoxic response to symbiotic seeds [28].

Other nonpathogenic endophytic fungi such as *Epichloe* spp. and lichen-forming fungi were also included. Symbiotic fungi, as a functionally diverse group of organisms, play an irreplaceable role in natural ecosystems by forming mutually beneficial symbiotic relationships with their hosts. From mycorrhizal fungi to endophytic fungi, from lichens to insect symbiotic bacteria, each class of symbiotic fungi exhibits unique structural characteristics and functional mechanisms.

It is worth noting that in the current research field, AM fungi are undoubtedly the most extensively studied group. Approximately 80% of terrestrial plant species establish mutualistic associations with these ancient fungal lineages, whose molecular, ecological, and evolutionary characteristics are well documented. However, our understanding of many symbiotic fungal groups, especially newly discovered ones, remains limited. Future research should focus on non-AM fungi, exploring their diversity, symbiosis mechanisms, and ecological functions, while continuing AM fungal studies. A comprehensive understanding of symbiotic fungal diversity will reveal their ecological roles and enable applications in conservation, agriculture, and biotechnology.

### 2.2. Effects of Symbiotic Fungi on Plant Growth and Development

Promotion of seed germination. ORM fungi facilitate orchid germination and development via enzymatic breakdown of seed coats [9]. While some fungi support seed germination, others promote seedling development [29]. Taxa such as *Tulasnella* and *Ceratobasidium* are critical for endangered orchids (e.g., *Epipectis flava*, *Dendrobium chrysotoxum*) [30,31].

Regulation of plant growth. Plants, including orchids, depend on symbiotic fungi for growth, with different fungal taxa performing specific functions. For example, *Tulasnella* strains enhance the growth of *Dendrobium chrysotoxum* seedlings [32], while endophytic fungi produce phytohormones such as IAA and GAs, regulating plant growth [33]. Optimal AMF spore levels promote biomass accumulation and improve growth in crops such as melon and sugarcane [34]. Maintaining a balanced fungal spore population is crucial for sustaining this beneficial symbiotic relationship.

Involvement in nutrient uptake. Symbiotic fungi alter the host plant’s root structure, expanding root absorption areas. AMF mycelium transports water to host cells, enhancing nutrient uptake. Plants inoculated with AMF show increased P, N, K, Ca, Mg, and Cu accumulation [35]. AMF’s phosphate transporters facilitate nutrient exchange between maize and wheat [36]. Endophytic and AMF fungi improve phosphorus uptake in Camellia oleifera [37].

Enhancement of plant defense. Fungal symbiosis boosts the host plant’s growth and resilience to biotic and abiotic stresses, for instance, enhancing tomato’s resilience to drought and salinity stress [38]. AMF aided *Wedelia trilobata*‘s growth in harsh conditions and bolstered its disease resistance [39]. Commensal fungi help plants resist pathogenic bacteria, with AMF reducing damage from *Tuta absoluta* in tomatoes [40]. Trichoderma enhances plant defenses against pathogens such as Aspergillus fumigatus and Fusarium [41].

Fungal symbiosis significantly influences plant life, enhancing plant nutrition, growth, and phytohormone regulation. As environmentally friendly pesticides, symbiotic fungi demonstrate potential for sustainable crop protection and offer innovative approaches to plant protection research.

Enhanced root action. Symbiotic fungi enhance productivity by strengthening roots. *Epichloe* and *S. indica* promote root proliferation through indole-3-acetic acid production [42]. Mycorrhizal fungi enlarge root absorption area and improve nutrient uptake efficiency, while symbionts induce broad-spectrum pathogen resistance via signaling pathways [43]. Endophytic fungi upregulate antioxidant defenses and stress-related genes, enhancing resistance to biotic/abiotic stresses, as seen in *Epichloe sinica*-colonized *Roegneria kamoji* [44,45]. *Epichloe*-associated bacteria further promote silicon accumulation, boosting stress tolerance [46]. The effects of symbiotic fungi on plant growth and development are multidimensional and complex, and their effects are not limited to nutrient absorption and stress resistance enhancement but involve the modulation of core aspects of plant physiology, metabolism, and defense mechanisms. Symbiosis fulfills essential growth requirements for both partners. However, outcomes depend on fungal species, plant genotype, and environment, sometimes causing growth inhibition or resource competition. These dynamics are governed by molecular interactions spanning multiple biological scales.

## 3. Role of Alternative Splicing in Plant–Fungi Symbiosis

AS regulation is orchestrated by an intricate network involving SR proteins, heterogeneous nuclear ribonucleoproteins (hnRNPs), and core spliceosomal components. SR proteins facilitate splicing through dual mechanisms: their RNA recognition motif (RRM) domains bind exonic splicing enhancers (ESEs), while arginine-serine-rich (RS) domains mediate interactions with U1 small nuclear ribonucleoprotein (snRNP) and U2 small nuclear ribonucleoprotein auxiliary factor (U2AF) [47]. Conversely, hnRNPs antagonize this process by binding intronic and exonic splicing silencers (ISSs and ESSs, respectively) and preventing spliceosome assembly at inhibitory sites [48]. The spliceosome itself, comprising U1 snRNP, U2AF, SF3B1, and other components, executes precise splice site selection through dynamic conformational rearrangements while maintaining strict splicing fidelity [49]. This regulatory network exhibits multilayered control through post-translational modifications (particularly phosphorylation), spatiotemporal expression patterns, and stress-responsive modulation. In plant and fungal systems, AS plasticity is further influenced by cell-autonomous metabolic states, environmental sensing pathways, and exogenous compounds that perturb either splicing factor expression or spliceosomal activity, demonstrating the remarkable adaptability of this regulatory system to both intrinsic and extrinsic cues.

### 3.1. Effects of Alternative Splicing on Plants

**Figure 2:**
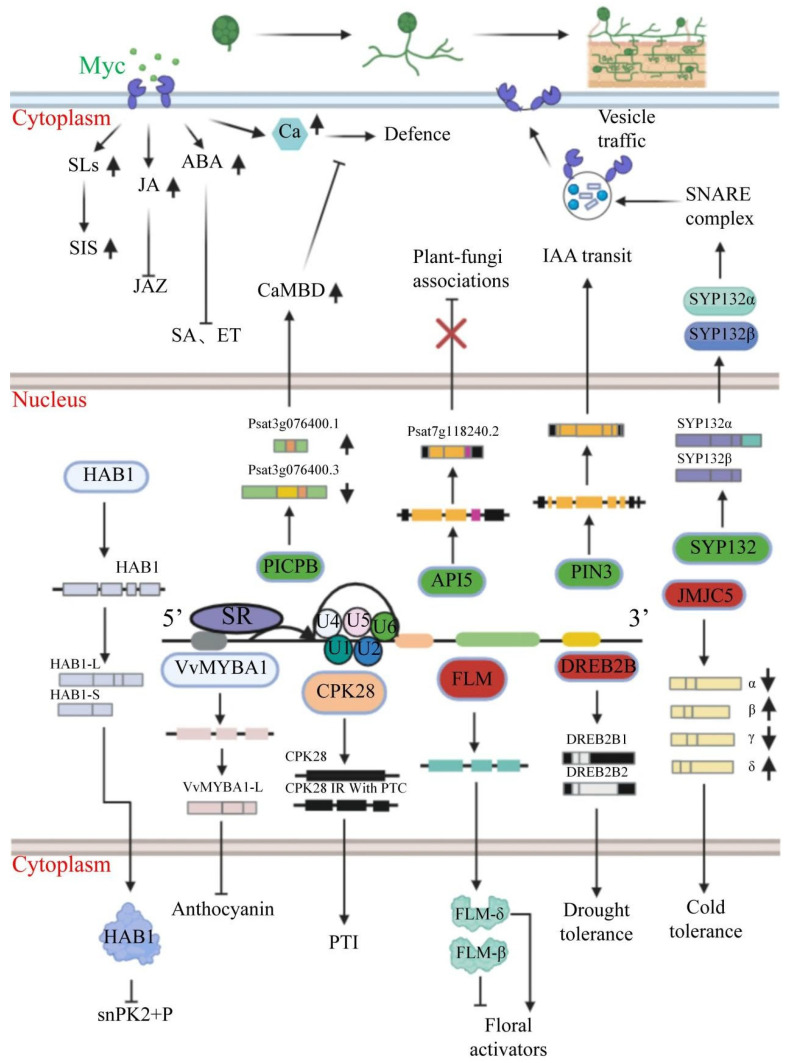
Mechanisms of AS regulation in plants under different irritant factors. The development of arbuscular mycorrhizae (AM) is broadly divided into four steps: (1) spore germination; (2) mycelial branching; (3) prepenetration apparatus (PPA) and hypha formation; and (4) root development [52]. AM fungi are recognized by host plant cells through the release of Myc, and hormones such as SLs, GA, and ABA are successively upregulated in plant cells during plant–fungus interactions. In these interactions, plants undergo specific AS, such as the upregulation of the isoform Past3g076400.1 with *PICPB*, encoding two calmodulin-binding structural domains (CaMBDs) [15]. The *API5* gene produces the longest isoform of the open reading frame (ORF), which is expressed in a targeted manner and facilitates root establishment with AM fungi [15]. Upregulated full-length PIN3 mediates root apex auxin transport to support mycorrhizal colonization [15]. The *SYP132α* isoform of *SYP13II* forms a complex with VAMP721d/e in the symbiont, delivering specialized cargo molecules to the symbiont, whereas the expression level of the *SYP132β* isoform has no effect on the symbiotic structure [18,53]. Specific alternative splicing also occurs in plants in response to external environmental stimuli. For example, ABA promotes the activation of *RBM25* and the removal of the last intron of *HAB1* to produce functional *HAB1* proteins that inhibit the phosphorylation of the SnRK2 protein kinase [54]. The *vvMYBA1-L* isoform of *VvMYBA1* interferes with anthocyanin synthesis in grapevine pulp [55]. *CPK28* mRNA produces an intron containing a premature termination codon (PTC) and thus generates a stronger defense response [56]. Temperature-dependent flowering genes (FLM) are also noteworthy. At low temperatures, *FLM-β* represses the activation of floral genes; at elevated temperatures, *FLM-δ* is upregulated and activates the transcription of floral genes [57]. *DREB2B* genes are highly expressed under abiotic stress conditions, and *DREB2B2* is highly expressed to improve plant stress tolerance [58]. *JMJC5* produces four isoforms: *JMJC5α* and *JMJC5γ* are downregulated under cold stress, while *JMJC5β* and *JMJC5δ* are strongly induced under cold stress [59].

AS regulates multiple plant processes through gene expression control, including flowering, stress responses, circadian clocks, and seed germination (Figure 2). AS influences temperature-dependent flowering by modifying protein isoforms and modulates phytohormone signaling pathways in response to stimuli to enhance heat, salt, and cold tolerance through specific gene variants [10,50]. Additionally, AS affects circadian rhythms, fruit ripening, and the regulation of the ABA signaling pathway during seed germination [13,51], highlighting its extensive impact on plant adaptation and development.

### 3.2. The Complexity of Alternative Splicing Regulation in Fungi

AS patterns in various eukaryotes share similarities. Fungal lifestyle shifts, from saprophytic to pathogenic, are influenced by host plant immune responses and soil phosphate levels driving fungal adaptations [60]. AS, including intron retention (IR), exon skipping (ES), alternative 5′ splice site (A5SS), and alternative 3′ splice site (A3SS), enable fungi to adapt to environmental changes, regulate growth and development, and influence pathogenicity and symbiotic interactions with plants [61]. Under stress conditions (e.g., host defense responses), IR can rapidly modulate the expression patterns of fungal-metabolism-related genes [62]. Alternative splicing through ES generates protein isoforms lacking specific functional domains, thereby regulating the host targeting of fungal effector proteins [63]. While A5SS and A3SS occur at relatively low frequencies during fungal infection, they exhibit specificity in key virulence genes (e.g., kinase and transporter genes) [62,64]. These splicing variants can influence the subcellular localization of effector proteins or their interactions with host targets, ultimately enhancing fungal infectivity.

The mutualistic interaction between fungal symbionts and their plant hosts is characterized by bidirectional molecular communication. Fungi benefit from plant-secreted SLs, enhancing their growth and metabolism [9], while plants respond to fungal Myc factors, adjusting their defenses for colonization [9]. Additionally, AS in symbiotic fungi, such as the RiCTR3A splice variant, may contribute to adaptations such as copper tolerance [21]. During the symbiosis between endogenous fungi and grasses, salicylic hydroxylase can help endophytic fungi degrade SA to escape plant immunity [65]. However, studies have found that there are multiple splicing subtypes of salicylate hydroxylases, many of which contain unspliced introns, which lead to gene frameshift and early downstream stop codons and ultimately produce nonfunctional salicylate hydroxylases [66]. Fungal symbiosis not only achieves parasitism on plants but controls the impact of other pathogens on plants. Therefore, although plant defense is weakened during the symbiosis process, nonfunctional proteins are produced through AS to ensure that plants still have basic defense capabilities against pathogens and finally achieve peaceful symbiosis. However, current research on AS in commensal fungi is still limited.

### 3.3. Involvement of Alternative Splicing in Fungi Interactions

AS plays dual roles in plant–fungal symbiosis: regulating host recognition mechanisms and sustaining mutualistic associations (Table 2). Studies have demonstrated that AS events dynamically regulate gene expression to facilitate plant–fungal environmental adaptation and symbiosis optimization through modulating symbiotic/immune signaling, material transport, and structural component formation.

Through mycorrhizal symbiosis, plants gain access to phosphorus and essential mineral nutrients via fungal networks [68]. In this process, AS affects the success of symbiosis and promotes the formation and function of symbionts by regulating the expression of genes related to nutrient absorption and signal transduction (such as API5 and PICBP) [15,69]. In addition, receptor kinases, cytoplasmic kinases, and genes such AS SYP132 and PIN3-like on the surface of plant cells regulate signal transmission and material transport through AS events, thus affecting the colonization and formation of symbiotic structures of fungi [15,18,70].

Second, AS events may also regulate metabolic pathways in plants during the interaction between plants and fungi. For example, during symbiosis with arbuscular mycorrhizal fungi, the lipid metabolism of plants, which is closely related to the composition and function of cell membranes, changes significantly [71,72]. Lipid synthesis and metabolism require the participation of multiple enzymes, and the gene expression of these enzymes may be regulated by AS events, thus affecting the stability and efficiency of the symbiotic relationship [73,74,75].

AS-mediated gene regulation in plant–endophyte systems maintains symbiotic balance [76] and facilitates nonpathogenic coexistence. Under stress conditions, AS events participate in maintaining a stable symbiotic relationship by regulating the expression of *CML21*, *CBL3*, splicing factor *SF3B5*, and *RiCTR3* genes in fungi [65,66]. In interaction with pathogenic fungi, AS events help plants recognize and resist infection by pathogenic fungi by precisely regulating the expression of defense-related genes and at the same time inhibit excessive defense response to symbiotic fungi, thus achieving a dynamic balance between symbiosis and pathogen defense [77,78].

In plant–fungus systems, alternative splicing could influence interaction outcomes by modulating small RNA pathways. sRNAs play a key role in transboundary communication, regulating metabolic pathways and defense responses in host plants [79,80]. Studies have shown that sRNAs of arbuscular mycorrhizal fungi may regulate specific sRNA of host plants through AS events, thus affecting the establishment and maintenance of symbiotic relationships [81,82].

In summary, AS events serve as a critical regulatory mechanism in the symbiotic relationship between plants and fungi. By modulating gene expression, metabolic pathways, and miRNA function, AS events can either promote or inhibit the establishment and development of symbiotic relationships. This regulation assists plants in adapting more effectively to environmental changes, optimizing symbiotic efficiency, and thereby enhancing their survival and adaptability. These findings not only offer novel insights into the molecular mechanisms underlying plant–fungus symbiosis but provide an important theoretical foundation for improving crop yield and stress resistance through the utilization of symbiosis in agricultural practices.

## 4. Molecular Mechanisms Associated with Fungus Symbiosis via Alternative Splicing

### 4.1. Molecular Mechanism of the Host’s Regulatory Factors

Mutually beneficial plant–fungal symbiotic interactions are highly beneficial to plant growth, development, and adaptation, making plant–fungal symbiosis a research priority for sustainable agriculture. Numerous studies have shown that the symbiotic relationship of fungi with plants triggers defense signaling pathways that are different from those of pathogenic bacteria, and these pathways can help symbiotic fungi colonize successfully. While plants allow symbiotic fungi to colonize, they also control fungal infection through kinases. In glycine max, the presence of TF GmNF-YA1a and GmNF-YA1b binds to the CCAAT sequence target; the GmNF-YA1a and GmNF-YA1b act as positive regulators to promote AMF colonization, and the expression of both IFs is downregulated by NARK in the root tissues of host plants under the influence of autoregulation to control AMF infection [83]. It has been found that NF-YA in plants is regulated by AS, and in M. truncatula, as the symbiont develops, about 50% of MtNF-YA1 undergoes AS in its 5′ leading sequence at the first intron of its 5′ leading sequence. However, the splicing pattern of MtNF-YA1 may be different from that of several other NF-YA family members [84].

### 4.2. Responsive Factors from Commensal Fungi

Symbiotic fungi establish a long-term relationship with the host plant throughout the life cycle of the fungus. Unlike necrotrophs, symbiotic fungi must overcome the plant’s defenses to develop within the host. An effector called SP7, which counters the plant’s immune response, was identified in Glomus intraradices. It was observed that SP7 has different cDNA sequences at various developmental stages in the fungi, with these cDNA sequences showing a high degree of similarity according to RT-PCR. By alternative splicing, five isoforms of SP7 were generated, and the mRNA isoform corresponding to a cDNA of 1.8 kb was the predominant form during symbiotic growth of the plant fungus (Table 2) [22]. Recent studies have revealed that symbiotic fungus-secreted SP7-like effector proteins (e.g., RiSP7 and GintSP7) can target host plant RNA-binding proteins (including SR proteins and hnRNPs), dramatically altering alternative splicing (AS) patterns of immunity- and symbiosis-related genes by modulating the subcellular localization and stability of splicing factors. For instance, Betz et al. demonstrated that RiSP7 directly binds host pre-mRNAs to promote critical exon retention in symbiosis-essential genes (e.g., *RAM2* and *PT4*) while inducing exon skipping in defense-related genes (e.g., *PR1*). This sophisticated AS reprogramming ultimately reshapes the plant’s hormone signaling network (particularly JA/SA balance) and carbon allocation metabolism (including lipid transport and glycolysis), providing novel insights into epitranscriptional regulation during symbiotic interactions [85,86].

## 5. Small Molecules and Their Target Proteins That Can Modulate Plant–Fungus Interactions

Small molecular compounds can act as signaling molecules and participate in the regulation of plant growth, adaptation to environmental changes, resistance to biological and abiotic stresses, the regulation of fungal symbiosis, and the life cycle of plants (Table 3).

Gibberellin (GAs) are mainly involved in regulating stem elongation and bud formation. Exogenous GA3 inhibits the expression of RAM1 and RAM2 [72], affects lipid transport, and inhibits the growth, development, and colonization of mycelia. AS a signaling molecule; it may regulate the AS event of gibberellin 2-β-dioxygenase (CsGA2ox8) in plants through feedback, controlling the plant to maintain a certain GA level [111], and the plant may also activate fungal symbiosis through GA inactivation [112].

As a phytohormone that inhibits plant growth, ABA promotes or inhibits the splicing of HAB1, resulting in changes in the relative content of different transcripts and ultimately affecting seed germination and postgermination development [113]. The differential expression of different CIPK subtypes regulates different signal transduction processes through binding with downstream gene promoters [114]. Moreover, in terms of plant stress resistance, ABA promoted the increase of different subtypes of OsGATA to different degrees in response to abiotic stress [115].

Jasmonate (JA) regulates plant growth, immunity, and environmental adaptation through root AM colonization and terpenoid metabolism modulation. While JA signaling restricts both pathogenic and symbiotic interactions [96], fungi such as Laccaria bicolor evolved countermeasures (e.g., MiSSP7-PtJAZ6 interaction) to promote symbiosis [116]. JAZ splicing variants regulate JA-lle levels and stabilize JAZ proteins during fungal adaptation [117]. JA also modulates AM symbiosis via phytohormone crosstalk [118], potentially enhancing fungal colonization in JA-insensitive plants.

Solanum lactones (SLs) are very important signaling molecules involved in plant–soil microbial interactions. Fungi can induce the auxin-related gene SL-IAA27 to control the synthesis of SL in plants by directly or indirectly regulating NSP1 [119] as a signal for fungal mycelium-directed growth, stimulating branching and growth of symbiotic fungal mycelium. During plant–fungus interaction, the symbiotic fungus releases Myc factor, which activates the plant’s symbiotic response; the host cell root SLs increase under CO stimulation, and the host root secretes SLs, which then stimulates the growth and branching of mycelia by activating the mitochondrial metabolism of the fungus, promoting the development of AMF [97]. SLs not only promote fungal symbiosis but inhibit the growth in many plants of pathogenic fungi and participate in plant defense [98]. At present, most studies have not directly shown that SLs are directly involved in the regulation of AS, but SLs may still indirectly participate in the regulation of AS through the regulation of phytohormones such as ABA [120].

Flavonoids are secondary metabolites of plants that promote the establishment of symbiosis by regulating the growth of fungi and the expression of symbiosis-related genes. AS regulates the biosynthetic and metabolic pathways of flavonoids (e.g., AS regulation of genes such as VvMYBA1 and DsCHS3) [55,121,122] and has also been found to affect symbiosis mechanisms found to be very similar to SLs [123]. Flavonoids, as crucial metabolic and signaling molecules, play a pivotal role in balancing plant immunity and symbiosis. They can target and bind to hnRNPs, thereby interfering with AS [124,125,126]. This suggests that flavonoids may not only regulate the establishment of symbiotic relationships but could indirectly modulate AS events of related genes by influencing splicing factors.

Lipids are also essential in symbiotic interactions between plants and fungi. During plant–fungus symbiotic interaction, lipids contribute to fungal mycelial growth and lateral branch formation [103], as well as secondary spore formation [103,105]. AMF use plant-secreted lipids for energy production through β-oxidation, the tricarboxylic acid cycle, and gluconeogenesis, supporting fungal growth and facilitating symbiosis [103]. Additionally, these lipids contribute to the development of membranes at the plant–fungal interface [103], playing a critical role in sustaining symbiotic structures. Ji et al. found that WRI is a key protein in plant lipid synthesis in castor seeds and that two splicing transcripts of RcWRI1 exist, of which RCWRI1-B is involved in enhancing the biosynthesis of fatty acids and oils during seed development [127]. Rich et al. also found that lipids in the rhizome were the main carbon source transmitted by plants to symbiotic fungi [128] and that the inactivation of WRI led to severe obstruction of fungal colonization, further demonstrating the importance of plant fatty acids for symbiosis between plants and fungi. The heterotopic differential expression of WRI splicing subtypes also participated in symbiosis at different developmental stages.

In addition to phytohormones, the plant signaling molecules NO and ROS, as important sources of nitrogen and important signals in the stress responses of fungi [129,130,131,132], not only participate in the regulation of symbiotic response but may affect the selective splicing of symbiosis-related genes by regulating the activity of splicing factors or epigenetic modification. Epigenetic modifiers, including trichostatin A (TSA) and 5-azacytidine, indirectly affect AS regulation of genes associated with symbiosis, such as TSA acting as a histone deacetylase (HDAC) inhibitor by altering chromatin structure and regulating splice factor expression or activity [133]. 5-azacytidine, a DNA methyltransferase inhibitor, may also be involved in influencing splicing factor expression or activity by reducing DNA methylation levels [134,135]. Natural products such as resveratrol and curcumin are also indirectly involved in influencing alternative splicing by regulating the expression and activity of splicing factors such as SRSF1 [136,137]. Secondary metabolites, flavonoids, promote the establishment of symbiosis by regulating the growth of fungi and the expression of symbiosis-related genes. sRNAs can not only help establish plant–fungus symbiosis but become regulators of AS through different molecular mechanisms [14,138]. lncRNA ASCO is regarded as an alternative splicing competitor that may interact with the spliceosome and inhibit the normal splicing process [139].

AS is a critical mechanism regulating gene expression and protein function during plant–fungus symbiosis. The studies reviewed herein indicate that small molecular compounds influence alternative splicing via a complex regulatory network, thereby optimizing the establishment and maintenance of symbiotic relationships. Future research will further elucidate the specific mechanisms of action of these small molecules and explore their potential applications in agriculture and ecological restoration.

## 6. Summary and Outlook

Plant–fungal symbiosis represents a widespread and reciprocal interaction in nature where plants and fungi form intricate associations through roots or other organs. AS modulates gene expression patterns that are critical for establishing and maintaining mutualistic interactions, thereby shaping the coevolution of plants and fungi. As a key regulatory mechanism in the evolution of plant–fungal symbiotic relationships, AS intricately coordinates various signal transduction pathways and influences metabolic activities.

The precise regulatory network of AS is orchestrated by splicing factors, spliceosome complexes, transcriptional kinases, and small-molecule compounds, which collectively determine the specific splicing patterns of pre-mRNAs to generate functionally diverse protein isoforms [140]. This sophisticated regulatory mechanism not only offers novel therapeutic targets (e.g., by the development of anticancer drugs by rectifying aberrant splicing of oncogenes) but demonstrates tremendous potential in agriculture. Targeted modulation of symbiosis-related AS events enables the design of novel biopesticides—for instance, small molecules that precisely modify splicing patterns of plant defense genes can simultaneously enhance symbiotic efficiency and reduce chemical pesticide usage. Current studies have verified that certain fungal-derived metabolites can dramatically reshape the AS landscape in host plants and significantly enhance symbiotic efficiency, thereby providing a molecular foundation for developing environmentally friendly agrochemicals.

AS fine-tunes plant symbiosis and stress responses by generating specialized isoforms of symbiotic and stress-related genes, particularly SYP132 and hormone signaling components. This molecular plasticity has been effectively harnessed in crop improvement—AS-based marker selection shortens breeding cycles, while CRISPR-mediated editing of splicing regulators such as SR proteins generates high-performance germplasm. Field-validated lines show dual benefits of increased yield and reduced fertilizer dependency, conclusively linking symbiotic efficiency with stress resilience and productivity. These findings establish a framework for sustainable crop optimization through RNA-level regulation.

Although significant gaps remain in AS research within plant–fungal symbiotic systems—particularly the lack of genome-wide symbiotic splicing atlases and identification of evolutionarily conserved regulatory elements—the scientific and translational value of this field is becoming increasingly prominent. Deciphering AS regulatory networks will yield breakthroughs in three dimensions: (i) at the fundamental level, elucidating the principles of RNA processing regulation in host–microbe interactions; (ii) technologically, developing spatiotemporal analytical methods integrating single-cell sequencing and nanopore technology; and (iii) in applications, creating intelligent molecular tools capable of coordinately regulating multiple symbiotic AS events. These advances will not only advance the concept of “precision symbiotic agriculture” but catalyze next-generation bioformulations that synergistically enhance crop resilience and symbiotic performance, offering innovative solutions for sustainable agricultural development.

## Figures and Tables

**Table 1:** Summary of articles of alternative splicing in plant–fungi symbiosis.

Plant Species	Fungi Species	AS Detection	References
*S. lycopersicum* cv.	*Rhizophagus irregularis*	AS profiling; RNA-Seq; PacBio (Novogene (Beijing, China)); Illumina (New England Biolabs, Ipswich, MA, USA)	[14]
*Pisum sativum* L.	*Rhizophagus irregularis*	AS profiling; bioinformatics; DESeq2; SUPPA2; DRIMSeq; IsoformSwitchAnalyseR (http://bioconductor.org/packages/IsoformSwitchAnalyzeR, accessed on 20 March 2025)	[15]
*Asparagus officinalis* L.	*Rhizophagus irregularis*	AS talavista; DESeq2; Bio-Rad (Bio-Rad CFX96 real-time PCR detection system, Boulder, CO, USA)	[16]
*Lotus japonicus*	*Glomus intraradices*	AS profiling; gene expression profiling	[17]
*Medicago truncatula* Jemalong A17; *O. sativa* ssp. japonica	*Rhizophagus irregularis*	AS profiling; Bio-Rad; gene expression profiling	[18,19]
*Solanum lycopersicum* L. culteugenic RioGrande 76R	*Glomus intraradices*	AS profiling; GMAP	[20]
*Daucus carota* L.	*Rhizophagus irregularis*	AS profiling; Bio-Rad	[21]
*Medicago truncatula* L.; Nicotiana benthamiana	*Glomus intraradices*	AS profiling; gene expression analyses	[22]

**Table 2:** List of host and fungus effectors in response to alternative splicing.

Alternative Splicing Gene	Fungus Name	Host	Cotyledon Type	Mechanism of Modulation/Determinant	Function	References
Plant Effector						
*PICBP*	*Rhizophagus irregularis*	*Pisum sativum* L.	dicotyledon	AS of the *PICPB* gene produces transcript variants containing either two or three calmodulin-binding domains (CaMBDs); the two-CaMBD isoform exhibits upregulated expression during arbuscular mycorrhizal symbiosis.	Symbiotic signaling	[15]
*AFC3*	*Rhizophagus irregularis*	*Pisum sativum* L.	dicotyledon	*AFC3* is involved in the alternative splicing process and regulates AS by regulating the expression of splicing factors, and isoforms with PTC are increased in the mycorrhizal roots.	Symbiotic signaling	[15]
*PIN3*	*Rhizophagus irregularis*	*Pisum sativum* L.	dicotyledon	AM symbiosis upregulates an alternatively spliced, truncated *PIN3* variant that mediates phytohormone transport.	Symbiotic signaling	[15]
*CAR11*	*Rhizophagus irregularis*	*Pisum sativum* L.	dicotyledon	In fungal symbiosis, *CAR11* full-length transcripts increase, whereas truncated or NMD-targeted transcripts decrease.	Symbiotic signaling	[15]
*ESP3*	*Rhizophagus irregularis*	*Pisum sativum* L.	dicotyledon	Longer-transcript isoforms containing HA2 or OB_NTP_bind regions are generated.	Symbiotic signaling	[15]
*P450*	*Rhizophagus irregularis*	*S. lycopersicum* cv.	dicotyledon	*P450* can produce three different isoforms through the AS process, but the expression of only one of these isoforms is upregulated in AMF colonization.	Symbiotic signaling	[14]
*CRK25*	*Rhizophagus irregularis*	*Pisum sativum* L.	dicotyledon	AM fungi activate plant defence responses but interrupt the transduction of plant-defence-related signals by causing premature termination codons in the ORF region of *CPK25* through the AS process, resulting in truncated isoforms, which are able to bind but not phosphorylate ligand signals, or degradation via NMD.	Immune regulation	[15]
*API5*	*Rhizophagus irregularis*	*Pisum sativum* L.	dicotyledon	The AM fungi, after symbiosis with plants, produce isoforms that retain introns as well as premature stop codons in the ORF region by AS to repress the expression of the *API5* gene.	Immune regulation	[15]
*GLP2-1*	*Rhizophagus irregularis*	*Asparagus officinalis* L. cv.	monocotyledon	Upregulation of isoform *GLP2-1* expression occurs in response to salt stress.	Immune regulation	[16]
*FTSH4*	*Rhizophagus irregularis*	*Pisum sativum* L.	dicotyledon	In AM symbiotic mycorrhizae, the Psat0ss1279g0480.1 isoform with more intronic fragments is upregulated, while the Psat0ss1279g0480.2 isoform is downregulated.	Immune regulation	[15]
*NUS1*	*Rhizophagus irregularis*	*Pisum sativum* L.	dicotyledon	Isoforms are produced that retain introns as well as premature stop codons in the ORF.	Sugar transport	[15]
*ENT3*	*Rhizophagus irregularis*	*Asparagus officinalis* L. cv.	monocotyledon	During salt stress, *ENT3* undergoes increased alternative splicing in mycorrhizal systems.	Substance transmembrane transport, immune regulation	[16]
*NAC068*	*Rhizophagus irregularis*	*Asparagus officinalis* L. cv.	monocotyledon	During salt stress, *NAC068* undergoes enhanced alternative splicing in mycorrhizal systems.	Metabolic regulation, immune regulation, phytohormone signaling	[16]
*AAP19-2*	*Rhizophagus irregularis*	*Asparagus officinalis* L. cv.	monocotyledon	Salt stress promotes alternative splicing of the *AAP19-2* gene in mycorrhizal plants.	Nutrient exchange, signaling, immune regulation	[16]
*CBL3*	*Rhizophagus irregularis*	*Asparagus officinalis* L. cv.	monocotyledon	Under saline stress, the *CBL3* gene shows significantly increased alternative splicing during mycorrhizal symbiosis.	Regulation of symbiosis and immune signaling pathways	[16]
*CML21*	*Rhizophagus irregularis*	*Asparagus officinalis* L. cv.	monocotyledon	Alternative splicing of *CML21* in mycorrhizae undergoes significant upregulation in response to salt stress.	Regulation of symbiosis and immune signaling pathways	[16]
*CYP21-1*	*Rhizophagus irregularis*	*Asparagus officinalis* L. cv.	monocotyledon	Upregulation of isoform *CYP21-1* expression occurrs in response to salt stress.	Regulation of symbiosis and immune signaling pathways	[16]
*ATGs*	*Rhizophagus irregularis*	*Asparagus officinalis* L. cv.	monocotyledon	*ATG* can produce multiple isoforms via AS, and the expression of the isoforms *ATG8I* and *ATG8C* undergoes upregulation in response to salt stress.	Nutrient cycling, immune regulation, maintenance of symbiotic structural homeostasis	[16]
*CER1*	*Rhizophagus irregularis*	*Asparagus officinalis* L. cv.	monocotyledon	Alternative splicing of *CER1* in mycorrhizae undergoes significant upregulation in response to salt stress.	Immune modulation, stability of symbiotic structures	[16]
*CB5LP*	*Rhizophagus irregularis*	*Asparagus officinalis* L. cv.	monocotyledon	Alternative splicing of *CB5LP* in mycorrhizae undergoes significant upregulation in response to salt stress.	Symbiotic signaling pathways, structural elements	[16]
*SYP13* *Ⅱ*	*Rhizophagus irregularis-infected Allium schoenoprasum*	*Medicago truncatula* Gaertn.	dicotyledon	*SYP13II* can produce two isoforms, *SYP13IIα* and *SYP13IIβ*, by alternative splicing. The *SYP13IIα* isoform is upregulated in AM-fungal-symbiotic plants and is involved in the transport of substances between plants and microorganisms.	Material transport, structural components	[18,19]
*DEAH1*	*Rhizophagus irregularis*	*Pisum sativum* L.	dicotyledon	Conformational changes in the spliceosome during its catalytic cycle are orchestrated by core splicing factors.	Structural components	[15,67]
*SF3B5*	*Rhizophagus irregularis*	*Asparagus officinalis* L. cv.	monocotyledon	A part of the U2 small nuclear ribonucleoprotein particle (snRNP), which participatesin alternative splicing, is formed.	Structural components	[16]
**Fungus Effector**						
*RiCTR3*	*Rhizophagus irregularis*	*Daucus carota* L.	dicotyledon	Mycorrhizal roots are highly expressed both *RiCTR3* splice variants (*RiCTR3A* and *RiCTR3B*); the first-intron-lacking *RiCTR3A* isoform enhances copper tolerance under copper toxicity.	Copper ion equilibrium, reactive oxygen species (ROS) stress adaptation	[21]
*SP7*	*Glomus intraradices*	*Oryza sativa L.*	monocotyledon	*SP7* can form isoforms of different lengths, and the isoform corresponding to the longest 1.8-kb cDNA is the predominant form during the growth of plant–fungus interactions.	Immune evasion, developmental regulation, metabolic coordination	[22]

**Table 3:** Summary of small molecules that affect plant–fungus symbiosis.

Treatment	Fungus Name	Host	Mechanism of Modulation/Determinant	References
GAs	*Rhizophagus irregularis*	*E. grandiflorum* cv. Pink Thumb	GA is involved in RAM1 expression through the CSSP signalling pathway and promotes AM fungal arbuscule formation.	[87]
	*Glomus irregularis*	*Solanum lycopersicum* L.	GA treatment inhibits fungal infection and arbuscule development in Arum-type mycorrhizae.	[88,89,90]
	*Paecilomyces formosus*	*Cucumis sativus* L.	Cucumbers synthesized nonfunctional GAs that reduced salt stress impacts.	[91]
IAA	*Laccaria bicolor*	*Populus tremula × Populus alba*	IAA helps ECM root development.	[92]
	*Paecilomyces formosus*	*Cucumis sativus* L.	Enhanced IAA production in cucumber plants contributes to salt stress tolerance.	[91]
SA	*Glomus intraradices*	*Nicotiana tabacum* L.	High-concentration SA treatment negatively affects fungal colonization.	[93]
Ethylene	*Glomus clarum*	*Solanum lycopersicum* L.	In epi plants, ethylene inhibits fungal colonization.	[94]
JA	*Glomus intraradices*	*Medicago truncatula* L.	JA biosynthesis promotes mycorrhization in G. intraradices.	[95]
	*Glomus intraradices*	*Solanum lycopersicum* L.	The JA signalling pathway limits symbiotic interactions between AM fungi and plants.	[96]
SLs	*Funneliformis mosseae*	*Medicago truncatula cv.*	Perception of strigolactones (SLs) by AM fungi leads to hyphal branching induction.	[97]
	*Mucor* sp.	*Arabidopsis thaliana* L.	SL mediates symbiosis through two mechanisms: extracellular signaling to AM fungi and intrinsic regulation in plants.	[98]
BRs	*Rhizoglomus irregularis*	*Solanum lycopersicum* L.	Mycorrhizal germination in plants is promoted through BR signalling.	[99]
ABA	*R. irregulare*	*Solanum tuberosum* L.	The stimulation of hyphal branching around spores by ABA suggests its positive effect on spore viability.	[100]
ROS	*Epichloe festucae*	*Lolium perenne* L.	NADPH oxidase (NoxA) inactivation mediates symbiotic transition by controlling fungal development in plant hosts.	[101]
2-Hydroxytetradecanoic acid (2-OH-C14:0)	*Gigaspora gigantea*	*Daucus carota* L.	Mycelial elongation and branching are stimulated.	[102,103]
Myristic acid (C14:0)	*R. irregularis*; *R. clarus* HR1; *G. margarita* K-1	*Daucus carota* L.	AMF are induced to form symbiotic spores.	[103,104,105]
miR393	*Rhizophagus irregularis DAOM197198*	*Medicago truncatula* Gaertn.	Impedes growth hormone signalling in AMF-containing host cells and affects tuft formation.	[106,107]
miR171h	*Rhizophagus irregularis*	*Medicago truncatula* Gaertn.	miR171h is involved in mediating the negative regulatory mechanism of *NSP2* to combat AMF overcolonization.	[82,107,108]
miR171b	*Rhizophagus irregularis*	*Medicago truncatula* Gaertn.	*LOM1* expression for mycorrhization is enhanced and protected.	[107,109]
RiCLE1	*R. irregularis DAOM 197198*	*M. truncatula* Gaertn.; *Pisum sativum* L.	Host root branching and AMF colonization are promoted.	[107,110]

## Data Availability

Not applicable.
